# Effect of Remote Ischemic Preconditioning on Acute Kidney Injury in Nondiabetic Patients Undergoing Coronary Artery Bypass Graft Surgery: A Secondary Analysis of 2 Small Randomized Trials

**DOI:** 10.1053/j.ajkd.2010.07.014

**Published:** 2010-12

**Authors:** Vinod Venugopal, Chris M. Laing, Andrew Ludman, Derek M. Yellon, Derek Hausenloy

**Affiliations:** 1 The Hatter Cardiovascular Institute, University College London Hospital, London, UK; 2 Centre for Nephrology, University College London, Royal Free Campus, London, UK

**Keywords:** Remote ischemic preconditioning, transient limb ischemia, coronary artery bypass graft surgery, acute kidney injury

## Abstract

**Background:**

Novel treatment strategies are required to reduce the development of acute kidney injury (AKI) in patients undergoing cardiac surgery. In this respect, remote ischemic preconditioning (RIPC), a phenomenon in which transient nonlethal ischemia applied to an organ or tissue protects another organ or tissue from subsequent lethal ischemic injury, is a potential renoprotective strategy.

**Study Design:**

Secondary analysis of 2 randomized trials.

**Setting & Participants:**

78 consenting selected nondiabetic patients in a university teaching hospital undergoing elective coronary artery bypass graft (CABG) surgery recruited to 2 previously reported randomized studies.

**Intervention:**

RIPC consisted of three 5-minute cycles of right forearm ischemia, induced by inflating a blood pressure cuff on the upper arm to 200 mm Hg, with an intervening 5 minutes of reperfusion, during which time the cuff was deflated. The control consisted of placing an uninflated cuff on the arm for 30 minutes.

**Outcomes:**

AKI measured using Acute Kidney Injury Network (AKIN) criteria, duration of hospital stay, in-hospital and 30-day mortality.

**Results:**

Numbers of participants with AKI stages 1, 2, and 3 were 1 (3%), 3 (8%), and 0 in the intervention group compared with 10 (25%), 0, and 0 in the control group, respectively (*P* = 0.005). The decrease in AKI was independent of the effect of concomitant aortic valve replacement and cross-clamp times, which were distributed unevenly between the 2 groups.

**Limitations:**

Retrospective analysis of data. More patients in the RIPC group underwent concomitant aortic valve replacement with CABG; although we have corrected statistically for this imbalance, it remains an important confounding variable.

**Conclusions:**

RIPC induced using transient forearm ischemia decreased the incidence of AKI in nondiabetic patients undergoing elective CABG surgery in this retrospective analysis. A large prospective clinical trial is required to study this effect and clinical outcomes in patients undergoing cardiac surgery.

Editorial, p. 1019

Acute kidney injury (AKI) affects up to 30% of patients undergoing cardiac surgery, with 1%-2% of patients going on to require dialysis therapy.^1^ Its presence is associated with significant morbidity and mortality, such that even after adjustment for patient comorbid conditions and surgical complications, the presence of AKI requiring dialysis therapy increases the risk of death by 8 times in this patient group.^2^ Furthermore, changes >0.5 mg/dL in serum creatinine level after cardiac surgery also contribute to a significant increase in mortality at 30 days postsurgery.^3^ A variety of treatment strategies have been investigated in an effort to decrease AKI incidence in patients undergoing cardiac surgery; however, on the whole, these have been disappointing (reviewed in^1^). Therefore, novel treatment strategies are required to decrease AKI incidence, preserve kidney function, and improve clinical outcomes in patients undergoing cardiac surgery.

In this regard, remote ischemic preconditioning (RIPC) may offer a novel noninvasive and virtually cost-free treatment strategy for decreasing AKI incidence in patients undergoing cardiac surgery (reviewed by Hausenloy and Yellon^4^). RIPC describes the phenomenon in which transient nonlethal ischemia and reperfusion applied to one organ or tissue protects another organ or tissue from a subsequent episode of lethal ischemia and reperfusion. Recent proof-of-concept clinical studies have shown that RIPC using transient ischemia and reperfusion of the lower limb can preserve kidney function in patients undergoing elective endovascular^5^ or open surgical repair^6^ of an abdominal aortic aneurysm. Whether RIPC is able to decrease AKI incidence in patients undergoing elective coronary artery bypass graft (CABG) surgery remains to be determined.

## Methods

### Patient Selection

**Figure 1:**
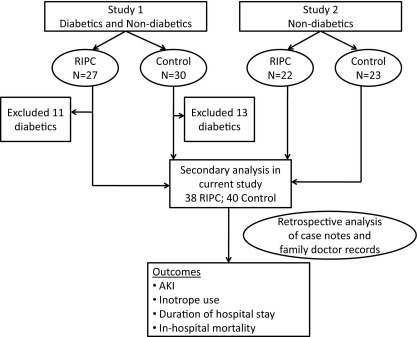
CONSORT (Consolidated Standards of Reporting Trials) flow diagram of patients included in the present study. Study 1 and study 2 refer to previously reported studies^7,8^ from which data were retrospectively analyzed for outcomes in the present study. Study 1 was registered at ClinicalTrials.gov as study number NCT00397163. Abbreviations: AKI, acute kidney injury; RIPC, remote ischemic preconditioning.

Ethics approval from the local University College London Hospital/University College London Ethics Committee was obtained. This study is a retrospective analysis of nondiabetic patients selected from 2 cohorts of patients undergoing CABG surgery who originally were recruited to investigate whether RIPC would decrease perioperative myocardial injury.^7,8^ Although the first study of 57 patients (30 control, 27 RIPC) included both diabetic (24 patients; 23 control and 22 RIPC) and nondiabetic participants undergoing CABG total,^7^ the latter study (45 patients: 23 control and 22 RIPC) included only nondiabetic patients. Therefore, to ensure uniformity, we included only the nondiabetic patients from the first study in this analysis, as shown in Fig 1. Family physician and medical notes for 78 nondiabetic patients (38 RIPC and 40 control) were analyzed retrospectively to investigate the effect of RIPC on kidney function, duration of hospital stay, and in-hospital mortality.

### Intervention: RIPC Protocol

RIPC consisted of three 5-minute cycles of right upper-limb ischemia induced by a blood pressure cuff placed on the right upper arm and inflated to 200 mm Hg, with an intervening 5 minutes of reperfusion during which time the cuff was deflated. Control patients had an uninflated cuff placed on the right upper arm for 30 minutes. The RIPC protocol was applied after anesthetic induction and before the start of surgery. As such, patients and cardiac surgeons were blinded to treatment allocation.

All nondiabetic patients previously randomly assigned to RIPC or control in 2 previously reported single-blind randomized controlled studies were selected. Randomization had been undertaken using computer-generated random numbers. Data were analyzed retrospectively for differences in AKI incidence, serum creatinine level, duration of hospital stay, and in-hospital mortality.

### Primary Outcome: Measurement of AKI

The Acute Kidney Injury Network (AKIN) recently proposed a new definition and classification of kidney injury that could be used as a uniform standard for patient management and clinical and translational research.^9^ This subsequently has been validated in the intensive care setting and correlated with outcomes in patients admitted to the intensive care unit.^10,11^ In our patients, AKIN criteria^9^ were used to define perioperative AKI stages 1, 2, and 3 during the first 72 hours.

### Statistical Analysis

Data are presented as mean ± standard deviation. Comparison between treatment groups was made using unpaired *t* test for continuous variables and χ^2^ or Fisher exact test for categorical variables. *P* < 0.05 is considered significant. Analysis was performed using SPSS statistical software, version 17.0 (SPSS Inc, www.spss.com). It is important to clarify at this stage that this analysis was retrospective and the end points used therefore were not prespecified. Therefore, no sample-size calculations are available for these data. Nevertheless, we proceeded with the analysis with a view to generating important pilot data for future studies specifically designed to measure differences in outcomes.

## Results

A total of 78 consented patients were randomly assigned to either RIPC (n = 38) or control (n = 40) before CABG surgery. Baseline characteristics were similar, although a small proportion of patients who underwent concomitant aortic valve replacement and CABG surgery were distributed disproportionately between the 2 groups, resulting in longer cross-clamp and bypass times in the control group (Table 1). Baseline preoperative troponin T levels were <0.01 μg/L in both treatment groups. Confirming findings of our previous study, RIPC decreased perioperative troponin T release during the 72 hours after cardiac surgery; total troponin T released, expressed as the area under the curve during the 72 hours after surgery, was 34.8 ± 28.7 μg/L in controls and 20.5 ± 10.2 μg/L with RIPC (mean difference, 14.3 ± 4.8 μg/L; 95% confidence interval [CI], 4.6-24.0; *P* = 0.005), a decrease of 41%.

The comparative primary outcomes are listed in Table 2. Fourteen patients (18%) developed AKI. Of 40 patients in the control group, 10 (25%) developed AKI stage 1 and none developed AKI stage 2 or 3. In contrast, only 1 of 38 patients (3%) in the RIPC group developed AKI stage 1, although 3 patients developed AKI stage 2. None of the patients in the cohort developed AKI stage 3 or required hemofiltration in the first 72 hours. The overall difference in AKI between the 2 groups was statistically significant (Pearson χ^2^, *P* = 0.005). There was no difference in duration of hospital stay. There were no in-hospital deaths, reflecting the low-risk nature of the cohort.

Because a greater proportion of patients in the control group underwent aortic valve replacement and had longer cross-clamp times, we corrected for the confounding effect of this imbalance by performing logistic regression analysis that included cross-clamp time, aortic valve replacement, and RIPC (vs control) in the regression model. RIPC resulted in a 10-fold decrease in the incidence of AKI stage 1 independent of concomitant aortic valve replacement (odds ratio [OR], 9.54; 95% CI, 1.12-81.29; *P* = 0.04). Aortic valve replacement (OR, 1.987; 95% CI, 0.395-9.985; *P* = 0.4) and cross-clamp times (OR, 1.014; 95% CI, 0.980-1.048; *P* = 0.4) were not independently related to AKI stage 1 in this cohort. The increase in incidence of AKI stage 2 in the RIPC group was not statistically significant.

When the analysis was repeated in the 67 patients (35 RIPC and 32 control) who underwent CABG alone, the difference in AKI incidence between the RIPC and control groups was still statistically significant. In the control group, 7 of 32 (22%) patients developed AKI stage 1 compared with 1 of 35 (3%) in the RIPC group (OR, 8.96; 95% CI, 1.03-77.66). In the RIPC group, 2 of 35 (6%) developed AKI stage 2 versus none in the control group. However, this difference was not statistically significant.

**Figure 2:**
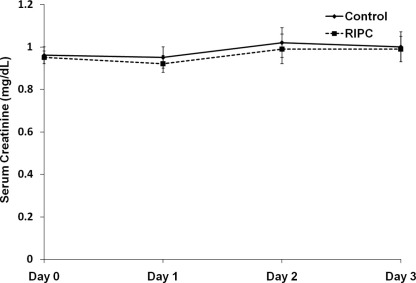
Graph shows trends in serum creatinine levels in the 2 groups at baseline and during 72 hours postoperatively. There was no significant difference between the 2 groups using analysis of variance with repeated measures. Note: Conversion factor for creatinine in mg/dL to μmol/L, ×88.4. Abbreviation: RIPC, remote ischemic preconditioning.

AKI was diagnosed using creatinine criteria in 10 of the 14 patients; in 1 case on day 1 and 9 cases on day 2 postoperatively. The creatinine level increase in these patients persisted for 48 hours. All these patients also had a decrease in urine output. All 14 patients with AKI fulfilled urine volume criteria (7 on day 1 and 7 on day 2 postoperatively), 10 of whom also had increases in serum creatinine levels persisting over 48 hours (AKI diagnosed and staged according to both criteria). Four patients who fulfilled urine volume criteria did not have a creatinine level increase during this period. Baseline and postoperative serum creatinine levels are shown in Fig 2. AKI was diagnosed on day 1 in 4 patients, on day 2 in 8 patients, and on day 3 in 2 patients.

## Discussion

In this retrospective analysis of nondiabetic patients undergoing elective CABG surgery, we found that RIPC using transient ischemia of the forearm decreased the incidence of AKI. This RIPC protocol previously has been reported to decrease perioperative myocardial injury incidence during cardiac surgery in adults^7,8^ and children^12^ and decrease both myocardial and renal injury incidence during surgery for endovascular^5^ and open surgical^6^ repair of abdominal aortic aneurysm.

Overall, there was a substantial decrease in the number of patients developing AKI (stages 1-3) in patients receiving RIPC before cardiac surgery. This was offset partially by an increase in AKI stage 2 incidence in the control group. However, the overall decrease in AKI incidence (14.5%) with RIPC was pronounced and warrants further investigation. None of the patients in our cohort developed AKI grade 3 or required hemofiltration postoperatively, probably because patients with pre-existing kidney failure and diabetic patients were excluded in the original studies. As such, the study group had a lower risk of developing AKI of all stages (including AKI stage 3) than a standard cohort of patients undergoing cardiac surgery.

In another clinical study of RIPC in patients undergoing abdominal aortic aneurysm repair, Ali et al^6^ showed that RIPC induced using intermittent iliac artery cross-clamping decreased the incidence of myocardial injury and infarction and renal injury (defined as serum creatinine >177 μmol/L). We have shown a similar beneficial effect of RIPC on decreasing AKI incidence during CABG surgery, although our definition of AKI was different from that used by Ali et al.^6^ We used a composite of changes in hourly urine output and serum creatinine measurements to define and stage the degree of AKI using AKIN criteria as opposed to a cutoff serum creatinine value alone.

The AKIN criteria^9^ were developed with a view to standardizing the measurement of AKI in the critical care setting. They were based on the finding that even small increases as low as 25 μmol/L in serum creatinine concentration in the first 24-48 hours increased mortality by an OR of 4.1.^13^ The AKIN also recognized the importance of decreasing urine output as a precedent of increasing serum creatinine levels and a sensitive and early discernible means of identifying patients with AKI.^9^ Because the AKIN criteria take into account even small changes in biochemical and functional indexes of kidney function, they are a more robust measure of AKI than cutoff values for serum creatinine level alone.

**Figure 3:**
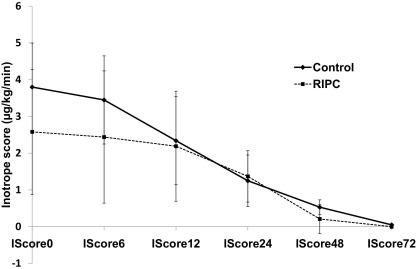
Graph shows serial inotrope scores during the 72-hour postoperative period (IScore followed by time in hours) in adult patients undergoing elective coronary artery bypass graft surgery. Compared with control, remote ischemic preconditioning (RIPC) has no effect on inotrope scores. Inotrope scores were calculated using the formula: Inotrope score = Dosages (in μg/kg/min) of Dopamine + Dobutamine + [(Adrenaline + Noradrenaline + Isoproterenol) × 100] + [Enoximone × 15], adapted from Ko et al.^15^ There was no significant difference between inotrope scores at different times using analysis of variance with repeated measures. Values presented as mean ± standard error of the mean.

Renal injury during cardiac surgery is believed to occur from several different injury pathways (reviewed in Bellomo et al^14^): exogenous and endogenous toxins, metabolic factors, ischemia and reperfusion, neurohormonal activation, inflammation, and oxidative stress. Our noninvasive RIPC protocol is likely to have direct effects on decreasing renal ischemia-reperfusion injury incidence as part of the systemic protective effect of this phenomenon. It is unlikely that a decrease in postoperative hypotension caused by myocardial stunning has a part because inotrope scores were similar between the 2 groups (Fig 3), and the antistunning effect of ischemic preconditioning intervention in general is not well established in the early phase.^16^

Whether RIPC using upper- or lower-limb preconditioning is different has not been directly compared. However, Loukogeorgakis et al^17^ have shown that remote ischemic postconditioning is effective only when the lower limb as opposed to the upper limb is used for the conditioning stimulus.

The systemic inflammatory response generated by cardiopulmonary bypass is well recognized.^18^ RIPC has been shown to decrease cardiopulmonary bypass–induced tissue injury.^19^ The same group also has shown a modification of inflammatory gene expression after RIPC in both murine models^20^ and humans.^21^ Specifically, genes involved in TNF (tumor necrosis factor) synthesis (MAPK [mitogen-activated protein kinase] and MAP/ERK [extracellular-regulated kinase]), TNF signaling, leukocyte adhesion, exocytosis, chemotaxis and apoptosis (caspase 8) were downregulated within 15 minutes and also 24 hours after the intervention, suggesting both an early and late anti-inflammatory effect.^21^ This was accompanied by upregulation of anti-inflammatory genes coding for heat shock protein 70 and calpastatin.^21^ It therefore is likely that the decrease in AKI incidence in our cohort is a result of a decrease in cardiopulmonary bypass–induced inflammatory response in addition to a protective effect against ischemia-reperfusion injury.

We have shown in this pilot study that RIPC may decrease perioperative AKI incidence in patients undergoing CABG. Because patients with diabetes and those with established kidney failure were excluded, this study group was at lower risk of AKI than a standard cardiac surgery cohort. It is reasonable to hypothesize that any protective effect of RIPC on kidney function may be even more pronounced in a higher risk cohort. Given the established risk that even mild AKI confers for in-hospital death, protective strategies to decrease AKI incidence after cardiac surgery (such as RIPC) may translate into decreased overall mortality.

Although this study has significant limitations because of its retrospective nature, it provides an early insight into the potential systemic benefits of RIPC in this clinical setting and builds a strong case for a larger study designed to measure differences in short- and long-term outcomes.

## Figures and Tables

**Table 1:** Baseline Variables

	Control (n = 40)	RIPC (n = 38)	*P*
Age (y)	66 ± 10	64 ± 10	0.5
Sex			0.6
Men	34 (85)	30 (79)	
Women	6 (15)	8 (21)	
Hypertension	22 (55)	29 (76)	0.05
Hypercholesterolemia	31 (78)	28 (74)	0.8
Myocardial infarction	9 (23)	9 (24)	0.9
Previous CVA	0 (0)	2 (5)	0.1
Peripheral vascular disease	2 (5)	1 (3)	0.6
Smoking			0.2
Current smoker	7 (18)	8 (24)	
Ex-smoker	14 (35)	18 (47)	
Never smoked	19 (48)	11 (29)	
Family history	20 (56)	14 (37)	0.2
Ejection fraction			0.9
Good (>55%)	35 (88)	33 (87)	
Fair (35%-55%)	4 (10)	4 (11)	
Poor (<35%)	1 (2)	1 (2)	
Serum creatinine (μmol/L)	84.24 ± 21.00	84.58 ± 15.69	0.9
EUROScore	2.9 ± 1.9	2.5 ± 2.2	0.4
NYHA class	1.8 ± 0.7	1.7 ± 0.7	0.7
CCS class	1.4 ± 0.8	1.6 ± 0.9	0.4
Preoperative medications			
Aspirin	28 (70)	24 (63)	0.5
β-Blocker	21 (53)	22 (58)	0.6
Statin	31 (78)	31 (82)	0.7
ACE inhibitor	25 (63)	26 (68)	0.6
Bypass time (min)	91 ± 33	80 ± 17	0.07
Cross-clamp time (min)	58 ± 29	45 ± 16	0.02
Myocardial preservation			0.4
ICCF	8 (20)	11 (29)	
Cardioplegia	32 (80)	27 (71)	
Concomitant AVR	8 (20)	3 (8)	0.1
Volatile anesthetics	24 (60)	24 (63)	0.6

*Note:* Values expressed as mean ± standard deviation or number (percentage). Abbreviations: ACE, angiotensin-converting enzyme; AVR, aortic valve replacement; CCS, Canadian Cardiovascular Society; CVA, cerebrovascular accident; ICCF, intermittent cross-clamp fibrillation; NYHA, New York Heart Association; RIPC, remote ischemic preconditioning.

**Table 2:** In-Hospital Outcomes

	Control (n = 40)	RIPC (n = 38)	*P*
AKI grade			0.005
1	10 (25)	1 (3)	
2	0 (0)	3 (8)	
3	0 (0)	0 (0)	
Dialysis	0 (0)	0 (0)	
SCr (μmol/L)			
Baseline	84.58 ± 15.69	84.24 ± 21.00	0.9
Day 1	84.23 ± 24.95	80.97 ± 25.39	0.6
Day 2	90.49 ± 36.75	87.63 ± 38.93	0.7
Day 3	88.68 ± 31.57	87.11 ± 38.51	0.8
Days in hospital	8.21 ± 4.62	8.59 ± 4.88	0.7

*Note:* Values expressed as mean ± standard deviation or number (percentage). Conversion factors for SCr (serum creatinine) in μmol/L to mg/dL, ×0.0113. Abbreviations: AKI, acute kidney injury (AKI Network criteria); RIPC, remote ischemic preconditioning.
